# Building on health security capacities in Indonesia: Lessons learned from the COVID‐19 pandemic responses and challenges

**DOI:** 10.1111/zph.12976

**Published:** 2022-05-26

**Authors:** Dewi Nur Aisyah, Chyntia Aryanti Mayadewi, Meiwita Budiharsana, Dewi Amila Solikha, Pungkas Bahjuri Ali, Gayatri Igusti, Zisis Kozlakidis, Logan Manikam

**Affiliations:** ^1^ Department of Epidemiology and Public Health, Institute of Epidemiology and Health Care University College London London UK; ^2^ Indonesia One Health University Network Depok Indonesia; ^3^ Faculty of Public Health Universitas Indonesia Depok Indonesia; ^4^ Ministry of National Development Planning (BAPPENAS) of the Republic of Indonesia Jakarta Indonesia; ^5^ Aceso Global Health Consultants Limited London UK; ^6^ International Agency for Research on Cancer World Health Organization Lyon France

**Keywords:** COVID‐19, health security, Indonesia, laboratories, pandemics, policy

## Abstract

As an active member country of the WHO's International Health Regulation and Global Health Security Agenda, Indonesia, the world's fourth‐most populous and largest archipelagic country has recorded the second‐highest COVID‐19 cases in Asia with over 1.8 million cases in early June 2021. This geographically and socially diverse country has a dynamic national and sub‐national government coordination with decentralized authorities that can complicate a pandemic response which often requires nationally harmonized policies, adaptability to sub‐national contexts and global interconnectedness. This paper analyses and reviews COVID‐19 public data, regulations, guidance documents, statements and other related official documents to present a narrative that summarizes the government's COVID‐19 response strategies. It further analyses the challenges and achievements of the country's zoonotic diseases preparedness and responses and lastly provides relevant recommendations. Findings are presented in four sections according to the Global Health Security Agenda capacities, namely epidemiological surveillance (detect capacity); laboratory diagnostic testing (respond capacity); data management and analysis (enable capacity); and the role of sub‐national governments. The COVID‐19 pandemic has been a catalyst for the rapid transformation of existing surveillance systems, inter‐related stakeholder coordination and agile development from the pre‐pandemic health security capacities. This paper offers several recommendations on surveillance, laboratory capacity and data management, which might be useful for Indonesia and other countries with similar characteristics beyond the COVID‐19 response, such as achieving long‐term health security, zoonoses and pandemic prevention, as well as a digital transformation of their governmental capacities.

**Impacts**

- Analysis of the health security capacities and responses provided in this paper might be useful for Indonesia and other resource‐restricted countries with diverse and large populations, complex geography or decentralized sub‐national authority.
- Multi‐sectoral coordination between government bodies, private sector and the community is very important in providing an emergency healthcare response that can be used in the future healthcare provision, such as to achieve long‐term health security and systemic digital transformation.
- Sufficient number of skilled human resources remain a pressing challenge to implement the planned and intended future responses on the ground.

## 1 INTRODUCTION

Health security is defined by the World Health Organization (WHO) as a set of proactive and reactive activities to minimize the impacts of acute public health events that endanger people's health across geographical regions and international boundaries (WHO, 2020a). The International Health Regulations (IHR) were established in 2005, providing a legal instrument governing the effective and timely response towards outbreaks and other health emergencies that may occur among countries (WHO, 2008, 2017).

To date, there are 196 member countries that are legally bound by the IHR, which requires them to report to the WHO about public health events (WHO, 2007), as well as establishing capacity for surveillance and response towards health emergencies, including potential outbreaks (WHO, 2020b). Indonesia joined the IHR since it came into force in 2007 and has taken part in global health security responses ever since (Ministry of Health Republic of Indonesia, 2011). Subsequently, the Global Health Security Agenda (GHSA) was established with 29 member countries in 2014, increasing to 65 in 2018 (Ministry of Health Indonesia, 2018). There are eleven GHSA action packages including zoonotic disease control; strengthening national laboratory systems; and workforce development, linking public health with law and multi‐sectoral rapid response, which are highly relevant to this paper (*Centers for Disease Control and Prevention*, 2014). Indonesia has been actively participating in the GHSA and was appointed as chair of the Steering Group in 2016 (WHO, 2016).

The coronavirus disease (COVID‐19) is a newly emerged infectious disease that has become a global pandemic impacting many aspects of everyday life (Abebe, 2020; Singhal, 2020). Since the first confirmed case in December 2019 in China, the total number of confirmed cases reached over 172.6 million, causing 3.72 million deaths worldwide by the beginning of June 2021 (WHO, 2021). In Asia, Indonesia has recorded the second‐highest number of COVID‐19 cases, after India, standing at a total of 1.85 million confirmed cases, and over 51 thousands deaths (idem).

Indonesia faces a unique context as an upper–middle‐income country (World Bank, 2019); it is the world's largest archipelagic country and fourth‐largest population, divided administratively into 34 provinces and 514 districts/cities, whose each sub‐national government has decentralized authority (Central Bureau of Statistics Indonesia, 2013). It also has 1331 ethnic groups, 2500 local languages and six recognized religions (Naim & Syaputra, 2011), all of which present a harmonization challenge for the deployment of any national campaign.

According to a recent report, approximately half of the countries reviewed globally had strong operational readiness capacities in place, in order to respond to potential health emergencies, such as the COVID‐19 pandemic (Kandel et al., 2020). In Indonesia specifically, the government responded systematically since January 2020 by creating standardized public health and clinical response protocols (MoH Indonesia, 2020d), which have been revised five times up to April 2021. These protocols aim to provide guidance on five sectors; however, this paper will only review the first three, where data are currently readily available. These sections are as follows: a) epidemiological surveillance; b) laboratory‐based response; and c) clinical and clinical data management (corresponding to detect, respond and enabling functions of the health security toolkit). Subsequently, the pre‐existing governmental preparedness towards zoonotic diseases is presented as a key element in relation to the health security perspectives and how the system evolved during the COVID‐19 pandemic, based on publicly available data, documents, regulations and statements. Capacity building and collaboration between different stakeholders are necessary as the world ‘builds back better’ in the post‐pandemic era. Thus, this manuscript also contains a short section on the role of local governments within Indonesia, their risk assessments and relative contributions, in particular concerning the implementation aspect of centrally disseminated guidelines.

## 2 DETECT CAPACITY: EPIDEMIOLOGICAL SURVEILLANCE

Implementation of surveillance—including during the COVID‐19 pandemic—is critical to limit disease spread and enable economic and social activities to resume as quickly as possible. Thus, efficient and effective laboratory capacity (Lippi & Plebani, 2020; Tang et al., 2020) coupled with high‐quality data—collated and interpretable—is necessary to support analyses, integrated across disciplines and across sectors.

The Indonesian Government's surveillance system is managed by the Ministry of Health (MoH), which has five surveillance sub‐systems: a) infectious diseases, b) non‐infectious diseases, c) health environment and behaviour, d) population health problem and e) health dimensions (MoH Indonesia, 2003). Since 2017, the MoH also has established a permanent Public Health Emergency Operating Centre (PHEOC) unit which is linked to the existing surveillance systems above to prepare for emergency health situations (MoH Indonesia, 2017a, 2019).

Prior to the COVID‐19 pandemic, the Indonesian government issued an instruction to national and sub‐national bodies to collaborate in detecting and responding to zoonotic diseases that can threaten national and global health security, including the routine human, animal and wildlife surveillance (President of Indonesia, 2019). Such zoonotic disease surveillance initiatives were of limited regional scope; however, their relative success at the local level set the tone for the subsequent pandemic response (Azhar et al., 2010; Hartaningsih et al., 2015).

Table 1 describes the chronological order of the MoH responses to the pandemic from early 2020, a month after the outbreak occurred in Wuhan, China. By mid‐January 2020, the national government already appointed 22 provinces to prepare the Early Awareness and Response System against COVID‐19. The first official guideline on clinical and public health COVID‐19 response was released on 28 January 2020 and then was revised five times in alignment with the WHO's guidelines.

**TABLE 1:** Chronological events of COVID‐19 responses in Indonesia (Ministry of Health Republic of Indonesia, 2020a)

Date	Event
31 December 2019	Pneumonia Cluster in Wuhan
15 January 2020	Preparation for the evaluation of the Early Alertness and Response System, as well as Early Alertness against COVID‐19 in 22 Provinces
28 January 2020	1st Edition of Novel Coronavirus (2019‐nCoV) Infection Preparedness Guidelines by the Ministry of Health (MoH) of the Republic of Indonesia
30 January 2020	WHO defined 2019‐nCoV as the Public Health Emergency of International Concern
31 January 2020	Organized COVID‐19 Hotline by the Health Crisis Center of the MoH
1 February 2020	Large‐scale data collection initiated for suspected cases of COVID‐19 by PHEOC
17 February 2020	2nd Edition of Guidelines for Preparedness for COVID‐19 by the MoH
24 February 2020	Implementation for the evaluation of the Early Alertness and Response System, as well as Early Alertness against COVID‐19 in 14 Provinces
2 March 2020	Indonesia reports two confirmed cases of COVID‐19
11 March 2020	WHO defines COVID‐19 as a Pandemic
March 2020	Implementation of Epidemiologic Investigation in Jakarta (capital city) and four cities nearby
11 March 2020	3rd Edition of Guidelines for Prevention and Control for COVID‐19 by the Indonesian MoH
16 March 2020	Assignment of the person in charge of the COVID‐19 Data Processing Team
27 March 2020	Issuance of Regulation of the Directorate General of Disease Prevention and Control MoH about Assignment of COVID‐19 Surveillance Team in Directorate General of Disease Prevention and Control (SK Ditjen P2P No. HK.02.02/I/1743/2020)
27 March 2020	4th Edition of Guidelines for Prevention and Control for COVID‐19 by the MoH
31 March 2020	Presidential Decree about Large‐Scale Social Restrictions due to the Acceleration of COVID‐19 Responses (PP No. 21 years 2020)
April 2020	Use of Surveillance Data Applications through the All Record TC‐19
April 2020	Procurement of VTM (Viral Transport Medium) and Swabs
27 April 2020	Online Coordination Meeting about Indicator of Early Alert and Response Systems in Pandemic Situation
18 May 2020	Online Coordination Meeting about Indicator of Early Alert and Response Systems in Pandemic Situation in five Provinces
7 May 2020	Releasing Surveillance Guidelines for Diseases with Vaccines in the COVID‐19 Pandemic Situation
4 June 2020	Training and dissemination of Epidemiologic Surveillance Indicators in Controlling COVID‐19
26 June 2020	Training and dissemination of Surveillance Data Application Usage through the New All Record TC‐19
June 2020	Procurement of VTM (Viral Transport Medium) and Swabs
June 2020	Recruitment of COVID‐19 Data Processing Human Resources in five Provinces
June 2020	Implementation of Technical Guidance for Monitoring and Evaluation and Validation of COVID‐19 Data
13 July 2020	5th Edition of Guidelines for Prevention and Control for COVID‐19 by the MoH
15–24 July 2020	Training and dissemination of the 5th Edition of Guidelines for Prevention and Control for COVID‐19, and Reinforcement of Daily Reporting System
July 2020‐Now	Implementation of Technical Guidance for Monitoring and Evaluation and Validation of COVID‐19 Data, as well as Continuing Evaluation of Early Alert and Response Systems in Pandemic Situation in eight Provinces

**FIGURE 1:**
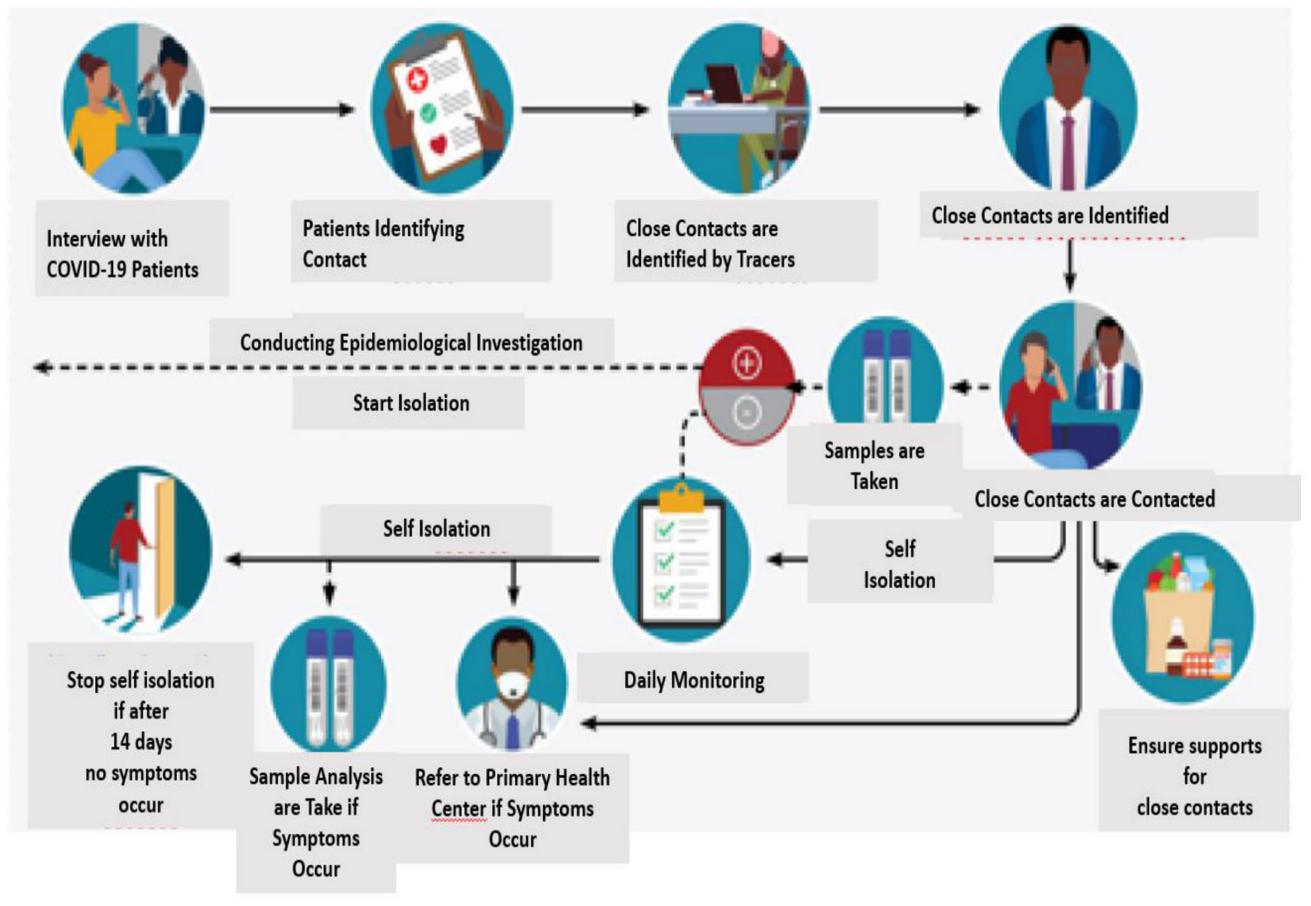
Contact tracing procedure in Indonesia (MoH Indonesia, 2020a)

Contact tracing and self‐quarantine are prominent aspects of outbreak detection and prevention (Keeling et al., 2020; Kucharski et al., 2020; Ruebush et al., 2021). In Indonesia, COVID‐19 contact tracing is sub‐nationally led and resource intensive. Specifically, by early November 2020, the total number of contact tracers across Indonesia reached over five thousand people (MoH Indonesia, 2020c). Figure 1 depicts the contact tracing process where the tracers submit a daily report with close contacts details through an online system, called ‘Silacak’, which was established in November 2020 (Bantu Daerah Deteksi COVID‐19, 2020).

However, three main challenges were observed. Firstly, data discrepancies occurred between national and sub‐national governments, even though the COVID‐19 information system for data recording was centralized into the New All Records (NAR TC‐19), developed by the MoH. This information misalignment was caused by (i) sub‐national laboratories that only sent reports to sub‐national health facilities but not to the MoH as well, leading to higher positive cases reported at a sub‐national as compared to the overall national level (Nugraheny, 2020); (ii) domicile location discrepancies (i.e. it contained the health facility's address, instead of the patient's) (Nurulliah, 2020); and (iii) staff unavailability in areas of high COVID‐19 transmission, resulting to reporting delays and some laboratories reporting only PCR‐confirmed positive cases instead of the total number of people tested (MoH Indonesia, 2020a).

The second challenge was the officers' capacity to collect and report real‐time data through surveillance systems. At the beginning of the pandemic, there was an insufficient number of trained, human resources to undertake the reporting tasks, with laboratory staff already overburdened with sample analyses, and many embedded within institutions monitoring zoonotic diseases, following slightly different laboratory protocols. Consequently, there was a variable completion rate for the initial sub‐nationally derived data, until staff numbers were consolidated. In addition, the initial version of the system recorded data in free text, making data input lengthy and data analysis difficult, before being streamlined and consistency of data input addressed in updated versions (Manafe, 2020; MoH Indonesia, 2020d).

Thirdly, the contact tracing reporting system development, where information was integrated centrally and became interoperable was unable to keep up with the sudden surge in cases during the first wave, although this was addressed in subsequent waves. As a result, some cases incurred delayed recording and reporting (MoH Indonesia, 2020a; Nugraheny, 2020). The exact impact of such a delay is difficult to quantify as it was time‐limited and only for some locations; however, it demonstrated that the scalability and flexibility of existing detection capacities could be further improved.

## 3 RESPOND CAPACITY: LABORATORY DIAGNOSTIC TESTING

Rapid and accurate laboratory diagnostic testing has been an essential response component to the current pandemic (Hendarwan et al., 2020; Kubina & Dziedzic, 2020; Shyu et al., 2020). Therefore, the Indonesian MoH regulated the COVID‐19 laboratory testing quality standards from the outset (MoH Indonesia, 2020b). In order to include new laboratories in the COVID‐19 testing network, sub‐national governments should propose a laboratory. An examiner then assesses it against MoH requirements and reports the outcome to the National Institute of Health Research and Development (NIHRD). Lastly, the NIHRD creates a network permit, allowing the said laboratory to input data into the centralized NAR TC‐19 data system. A similar approach was used successfully in Indonesia for the establishment of a surveillance laboratory network for Avian influenza from 2014 onwards (Hartaningsih et al., 2015).

**FIGURE 2:**
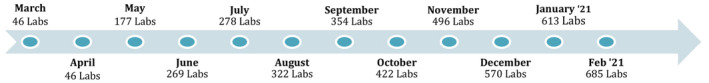
Increasing number of COVID‐19 referral Laboratories in Indonesia (National Institute of Health Research and department MoH Indonesia, 2021)

Figure 2 Increase in the number of COVID‐19 referral laboratories in Indonesia. The first laboratory was the NIHRD laboratory in the capital, DKI Jakarta. However, it was only able to handle samples from nearby cities. By collaborating with other institutions, NGOs and international donors, Indonesia, was able to increase their referral laboratories network to 685 across all 34 Indonesia's provinces as of February 2021, although the response capacity still has variability due to the differences in existing equipment infrastructure. For example, 558 diagnostic laboratories are equipped with RT‐PCR, 70 with molecular rapid test (TCM) and 57 with RT‐PCR and TCM equipment (Hendarwan et al., 2020). The largest numbers of laboratories are in the provinces of West Java (104 laboratories), DKI Jakarta (97 laboratories) and East Java (90 laboratories) (NIHRD, 2021), reflecting the areas of higher population density and larger clinical needs.

Indonesia showed a weekly increase in COVID‐19 testing capacity with a rapid increase from September 2020 during the second wave (MoH Indonesia, 2020). As of mid‐January 2021, Indonesia successfully fulfilled and consistently complied with the WHO's testing standard which is getting 1000 people tested per one million people).

However, three main challenges remain: Firstly, laboratories are unevenly distributed across provinces. Some provinces have large numbers of diagnostic laboratories such as DKI Jakarta (97), East Java (90), West Java (104), while others have far fewer, such as Aceh (six), North Maluku (seven), Southeast Sulawesi (five) and Gorontalo (two). While this is partially explained by the relative population density and clinical needs, this uneven distribution may lead to testing capacity asymmetry in the long‐term, hampering health security efforts.

Secondly, a limited number of qualified, laboratory‐trained staff can hinder operations even in areas where equipment and reagents are sufficiently provided (MoH Indonesia, 2020f). Thirdly, the oversight alignment and standardization challenge in implementing guidelines across national and sub‐national structures (Doni Monardo Ungkap Ego Sektoral, 2020) because participating laboratories are supervised by eleven different ministries/government institutions, sub‐national government agencies and private sector facilities (MoH Indonesia, n.d.)

## 4 ENABLING CAPACITY: DATA MANAGEMENT AND ANALYSIS

Epidemiological data, such as the incubation time and transmission dynamics of pathogens, allow the creation of appropriate reference datasets for ongoing pandemic response decision‐making (Ma, 2020; Rybniker & Fätkenheuer, 2020). It is then crucial to collect, analyse and share this information in a timely manner (Modjarrad et al., 2016).

**FIGURE 3:**
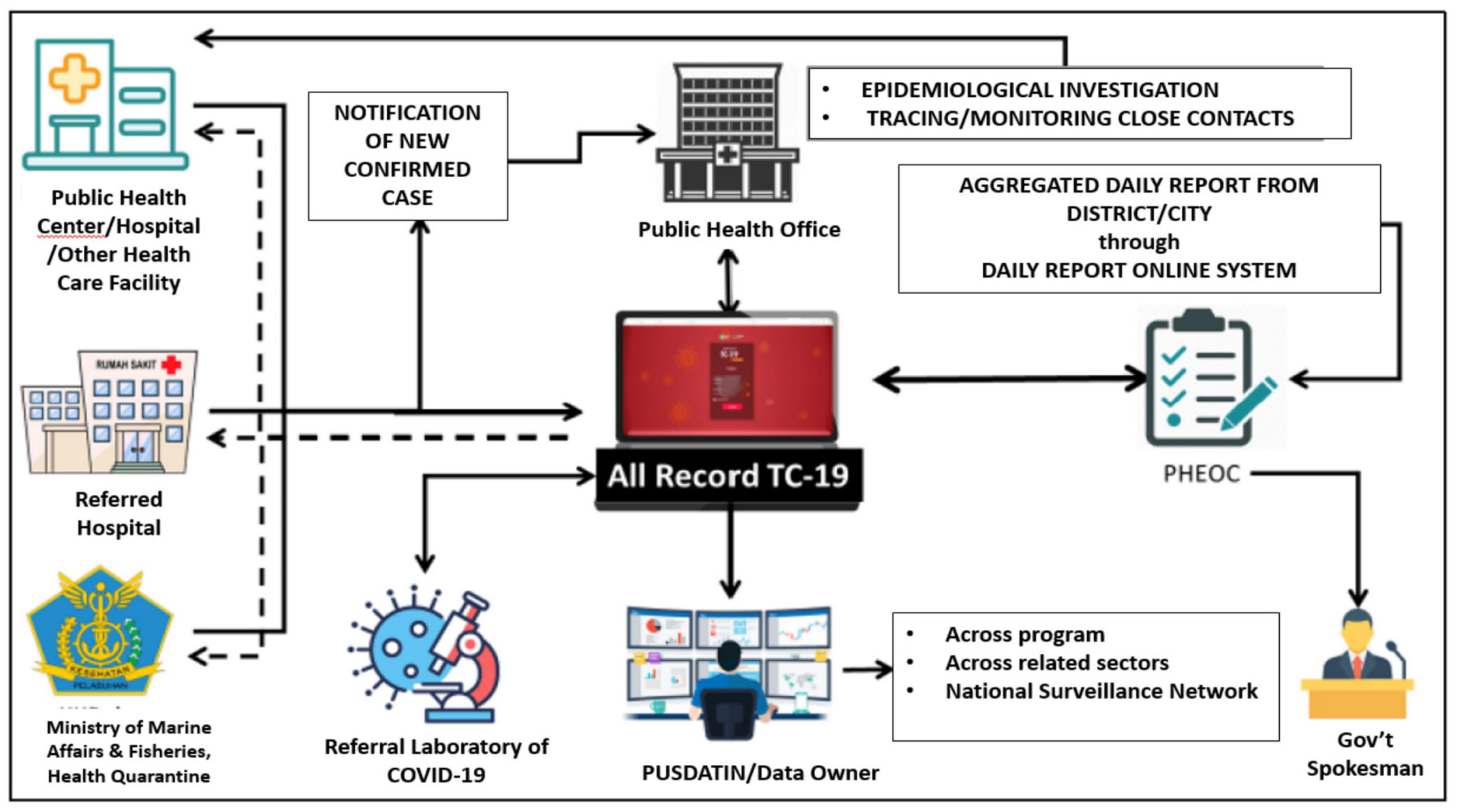
Flow of data reporting and recording

Various sources of COVID‐19 data emerged from mobile applications developed independently by the MoH, sub‐national governments and other institutions at the start of the pandemic. In order to avoid this data fragmentation, the NAR TC‐19 information system was developed by the MoH Pusat Data dan Informasi (Pusdatin—Information and Data Center) as the main, centralized national reporting toolkit used to collect laboratory data, as well as cases verification/confirmation. The Pusdatin integrates all COVID‐19 data nationally supported by the MoH data centre and data warehouse infrastructure. As Figure 3 shows, the role of Pusdatin is not restricted to COVID‐19, but can extend to include a wider set of health data. The pandemic acted as a catalyst for Pusdatin to create a central data processing architecture from all related MoH bodies. For example, the NAR TC‐19 organizational system has now been integrated with the Tuberculosis Information System and sub‐national information systems in Central Java, West Java and DKI Jakarta provinces. Therefore, Pusdatin is evolving to provide an integrated national reference point and governmental toolkit for public health which did not exist before, though some systems were tested for their cross‐sectoral linking as case studies in previous years as part of zoonotic disease surveillance (Setiawaty et al., 2015).

There are additional mobile applications developed for field surveillance, such as PeduliLindungi by the Ministry of Telecommunications and Informatics. This can help relevant government agencies carrying out contact tracing similar to UK's Bluetooth‐enabled ‘track and trace’ application (O'Dowd, 2020), which relies on community participation to share and register location data, while notifying users if they are in a red zone. Such applications can be enabled locally, according to the emergence of subsequent outbreaks, yet linked under a central national umbrella.

The National Task Force for the Acceleration of Handling COVID‐19 developed the Bersatu Lawan COVID‐19 (BLC) mobile application that integrates surveillance data and policies across governmental sectors. These are interoperable, shown in real‐time, while data received by the Task Force can be directly monitored by other national and sub‐national government bodies. In September 2020, BLC was upgraded to BLC‐Next Generation with the main concept of integrating cross‐sectorial data (health, transportation/mobility, public communication and socio‐culture), including to monitor community compliance to health protocols. It is envisaged that machine learning and artificial intelligence will be used to perform deep analysis and evaluate the development of COVID‐19 cases, as well as the effects of implemented public health measures. This is a crucial and novel big‐data capacity development in Indonesia that allows the transparent cross‐sectoral review of governmental guidelines and their subsequent implementation within a sectoral context.

Three challenges are identified. Firstly, the systems integration of many different data sources; since prior to an integrated national registration system (NAR TC‐19), many sub‐national governments developed their own systems. After the NAR TC‐19, those datasets needed to be extracted and re‐integrated into the national system. This caused an additional strain on resources and concerns over data quality (Nashr, 2020; West Java Provincial Government, 2020).

Secondly, the lack of trained staff was evident in provinces where data input was immediately needed. This occurred across different operational levels, including front‐line and quality controller levels (MoH Indonesia, 2020a). Thirdly, system interoperability due to institutional and geographical fragmentation remains critical for the Indonesian archipelago, where governmental agencies are often replicated across different locations. Thus, the independent creation of datasets can take place from different governmental departments, or from the same department across widely geographically disconnected regions. In addition, this fragmentation is reflected in the extant infrastructure, with some regions having much better‐enabling capacities than others. This is most appreciated when a real‐time collection of data is implemented, as slow access and electricity blackouts can disrupt the reporting (Internet Di Papua Mati, 2021; Taher, 2020).

## 5 THE ROLE OF SUB‐NATIONAL GOVERNMENTS

During the 2012 avian influenza (H5N1) outbreak, the Indonesian MoH involved sub‐national governments through a series of consultations to formulate prevention strategies and integrated surveillance of poultry and humans; raising awareness of zoonotic diseases; increasing referral hospital and laboratory capacity; simulated avian influenza response; as well as strengthening research and the One Health system (MoH Indonesia, 2012). These plans were put to the test in 2017 during a limited anthrax outbreak. As a result, the national government mandated the cooperative work of inter‐related sectors and maintained a good sub‐national government coordination (MoH Indonesia, 2017b; President of Indonesia, 2019) laying the foundation for the extensive coordination during the pandemic. The importance of these sub‐national stakeholders became quickly evident, in particular in organizing front‐line responders and in the implementation of the surveillance systems, data management and laboratory capacities mentioned previously.

As part of the COVID‐19 response, sub‐national governments ensured sufficient numbers of field health officers, logistics support and infrastructure, and also established an official command line for reporting. Sub‐national governments also controlled data management processes to ensure completion and compliance, in addition to data reporting. Positive feedback mechanisms, such as limited monetary incentives, were also deployed in rare occasions to encourage implementation and compliance (Badan Nasional Penanggulangan Bencana [BNPB—Indonesian National Agency for Disaster Countermeasures], n.d.; Koagouw, 2020). Sub‐national governments were tasked to quality check, monitor and evaluate the submitted reports to prevent any potential data errors so that specific epidemiological zones can be determined, and appropriate actions taken. This was an efficient mode of operation, as it ensured that a general roll‐out of guidelines could be achieved to a national standard, while taking into account local specificities and capacities.

Moreover, sub‐national governments were able to undertake laboratory, human resources, infrastructure and facility mapping to help the national government measure pandemic response capability. For instance, DKI Jakarta showed a rapid COVID‐19 response, based on an unprecedented public–private collaboration by maintaining a 97‐strong public and private laboratories network by February 2021. Private sector participation was critical in increasing testing and tracing capacity, as facilitated by local knowledge (Aisyah et al., 2021). In parallel, DKI Jakarta developed an integrated information system that included not only the number of confirmed cases, but also the capacity of hospital beds, distribution maps and area control maps (Government of DKI Jakarta Province, n.d.).

Several other provinces showed similar initiatives, customized to their needs. The Central Java province developed the Jogo Tonggo (Saving Neighbours) application to eradicate COVID‐19, utilizing a community‐based approach. Jogo Tonggo empowers residents for active participation to protect each other from COVID‐19 transmission through citizens associations (Government of Central Java Province, 2020). Jogo Tonggo also allows citizens to report any social problems related to the pandemic (Government of Surakarta City, n.d.).

However, the following challenges emerged within Indonesia's complex decentralized government authorities (Hill & Vidyattama, 2016; Von Luebke, 2007). Firstly, the variation in sub‐national capacity due to the decentralized authority and fiscal ability resulted in differences in capacity and human resources between provinces, cities and districts (Lebang et al., 2021). Secondly, the reporting systems developed independently by different sub‐national governments, persisted and had to be reorganized rapidly and with a substantial resource strain in order to streamline into a national reporting system, facilitating a national‐level analysis (MoH Indonesia, 2020a; Nashr, 2020; West Java Provincial Government, 2020). Thirdly, highly varied geographic conditions and different levels of population mobility between rural and urban areas had a significant influence in the implementation rate of the pandemic control measures, in some cases resulting to different interventions and comprehension (BNPB, 2021). For instance, DKI Jakarta introduced a set of measures called PSBB (Tim DetikCom, 2020), while its neighbouring province, West Java, introduced a different policy called PSBM. These different names, abbreviations and measures caused confusion, especially for those who commute between provinces (Shalihah, 2020).

## 6 DISCUSSION

This paper provides a detailed narrative view of Indonesia's COVID‐19 pandemic responses, as relating to the health security capacities. Although the overall pandemic response was considered proportional to the magnitude of the public health pressure (Aisyah et al., 2020) there were gaps and challenges identified in handling the pandemic and these can be summarized in Table 2.

**TABLE 2:** Summary of challenges and recommendations to build health security capacities in Indonesia

Health security capacity	Challenges	Recommendations
Detect capacity	Data discrepancies between national and sub‐national governments Officers' capacity to collect and report real‐time data through surveillance systems Capacity of national tracing reporting system to keep up with sudden surge of cases	A balance coordination between centralized national approach and accommodating sub‐national dynamics
Respond capacity	Uneven distribution of laboratories across provinces A limited number of qualified, laboratory‐trained human resources Oversight alignment and standardization across national and sub‐national government structures	Horizontal collaboration across laboratories, owned by different governmental agencies Enhance sub‐national government's contribution to mobilize human resources as volunteers
Enabling capacity	Systems integration of many different data sources and data warehouses The lack of trained human resources for data input Systems interoperability due to both the institutional and geographical fragmentation	Shifting the view from time‐bound project into a long‐term commitment towards digital transformation Enhancing the role of sub‐national governments in utilizing and disseminating information

Regarding the capacity to detect, it is evident that previous infectious disease emergencies in Indonesia, including the 2002 SARS‐CoV, 2009 H1N1 influenza pandemic and others, have strengthened the overall need for continuous surveillance. In particular, zoonotic disease surveillance initiatives provided a fertile ground for different approaches to be tested and improved within the Indonesian context. In the current pandemic, the most effective model appears to be one that is coordinated nationally by the MoH and implemented at national and sub‐national level, by national and sub‐national agencies and relevant stakeholders, using a system that is transparent and allows controlled access of information. This has proven effective in multiple settings for COVID‐19 (Crooks et al., 2020; Nunziata et al., 2020) as well as for other disease outbreaks (Barbadoro et al., 2020; McGinnis et al., 2020). More centralized systems might detract from an established sub‐national capacity in an effort to respond to pandemics via tertiary level healthcare (Banatvala, 2020). Thus, a balanced approach is needed, as the role of the sub‐national government within the Indonesian context remains critical, in verifying and monitoring real‐time cases, and implementing public health policies.

Regarding the capacity to respond, there were severe challenges in the scaling up of the response as the extent of the pandemic became apparent. The challenges included the availability, recruitment and training of staff; guaranteeing the availability of reagents and the adequacy of personal protective equipment (PPE); and capacity sharing within the laboratory network. These challenges were universal and not specific to Indonesia (Bhattacharya et al., 2020; Rasmussen et al., 2020). The effective response within the Indonesian context came from coordinating the agglomeration of different diagnostic laboratories under a single network umbrella. This approach was successful, however presented integration and harmonization challenges in turn. In effect, this was a similar approach as to the sub‐national governments described previously, except the collaboration took place horizontally, that is across governmental departments and agencies. This policy has similarities to the recommendations successfully followed in other settings, where a rapid surge in capacity was needed (Carenzo et al., 2020; Sparks et al., 2021). Particular mention needs to be made to the contribution of sub‐national governments who were able to mobilize, train and mentor additional laboratory staff and community volunteers for data input, tracking and tracing (MoH Indonesia, 2020e).

In order to enact an enabling capacity, the agile flow of data and reporting is required. Beyond infrastructural needs, any system would need to be query‐able by end‐users and connected to networks nationally. This requires interoperability across institutions and sectors in national and sub‐national levels. This is urgently needed for pandemic surveillance (Buckee, 2020) and is viewed as a long‐term commitment towards the digital transformation in Indonesia. As the national government continues to strengthen the management and real‐time flow of centralized data, sub‐national governments find themselves as both data generators and end‐users. Thus, it is critical to define the role of sub‐national governments in the utilization and dissemination of comprehensive, accurate information.

## 7 LIMITATIONS

This manuscript has certain limitations, while it provides a detailed view of the Indonesian response to the current pandemic, the major source of information are the different departments of the Indonesian government. There is the possibility that additional information might remain within sub‐national governments and/or within specific agencies; however, that information can be difficult to access. An additional limitation of a single‐major source is that of an inherent reporting bias. While the response to the pandemic has remained largely successful for the time period described (as compared to other highly populated nations in the same region of the world), the authors have made a particular effort to include the challenges highlighted by the different sources in an effort to address this bias.

## 8 CONCLUSION

This paper provides a detailed narrative view of Indonesia's COVID‐19 pandemic responses viewed through the lens of health security capacities. Despite the number of challenges highlighted, there has been an organized and controlled response, based on the lessons learned from past zoonotic surveillance initiatives, with unprecedented levels of systems integration in the history of the Indonesian government. Further strengthening of these capacities would be needed in order to control the current as well as preventing future zoonotic disease outbreaks. The lessons learned would need to inform the future design of the emergency response mechanisms if substantial progress is to be achieved.

According to the WHO, high‐level operational readiness will allow a timely, effective and efficient response. Achieving readiness is a continuous process of establishing, strengthening and maintaining a multisectoral response infrastructure that can be applied at all levels, which follows an all‐hazard approach, and which focuses on the highest priority risks. Operational readiness builds on existing capacities to design and set up specialized arrangements and services for an emergency response.

## CONFLICT OF INTEREST

None to be disclosed. While two authors are affiliated with Aceso Global Health Consultants Ltd, which is a private company, we declare that this research project does not receive funding from Aceso Global Health Consultants. The company does not have a role in the study design, data collection and analysis, decision to publish or preparation of the manuscript. Dr. Logan Manikam is the director of the company, and Gayatri Igusti is an employee of the company. However, both of them contributed to this paper on a pro‐bono basis.

## DISCLAIMER

Where authors are identified as personnel of the International Agency for Research on Cancer/WHO, the authors alone are responsible for the views expressed in this article and they do not necessarily represent the decisions, policy or views of the International Agency for Research on Cancer/WHO.

## ETHICAL APPROVAL

There was no ethics approval required for this study, as the data have been released publicly by the government of Indonesia.

## Data Availability

The data that support the findings of this study are openly available in Satgas COVID‐19 Republik Indonesia at https://covid19.go.id/.
